# Microbially Induced Calcium Carbonate Precipitation as a Bioremediation Technique for Mining Waste

**DOI:** 10.3390/toxics12020107

**Published:** 2024-01-27

**Authors:** Samantha M. Wilcox, Catherine N. Mulligan, Carmen Mihaela Neculita

**Affiliations:** 1 Department of Building, Civil and Environmental Engineering, Concordia University, Montréal, QC H3G IM8, Canada; 2 Research Institute on Mines and the Environment (RIME), University of Quebec in Abitibi-Témiscamingue, Rouyn-Noranda, QC J9X 5E4, Canada; carmenmihaela.neculita@uqat.ca

**Keywords:** mining waste, bioremediation, MICP, precipitation, CaCO_3_, urease

## Abstract

Mining waste represents a global issue due to its potential of generating acidic or alkaline leachate with high concentrations of metals and metalloids (metal(loid)s). Microbial-induced calcium carbonate precipitation (MICP) is an engineering tool used for remediation. MICP, induced via biological activity, aims to precipitate calcium carbonate (CaCO_3_) or co-precipitate other metal carbonates (MCO_3_). MICP is a bio-geochemical remediation method that aims to immobilize or remove metal(loid)s via enzyme, redox, or photosynthetic metabolic pathways. Contaminants are removed directly through immobilization as mineral precipitates (CaCO_3_ or MCO_3_), or indirectly (via sorption, complexes, or inclusion into the crystal structure). Further, CaCO_3_ precipitates deposited on the surface or within the pore spaces of a solid matrix create a clogging effect to reduce contaminant leachate. Experimental research on MICP has shown its promise as a bioremediation technique for mining waste. Additional research is required to evaluate the long-term feasibility and potential by-products of MICP-treated/stabilized waste.

## 1. Introduction

**Figure 1:**
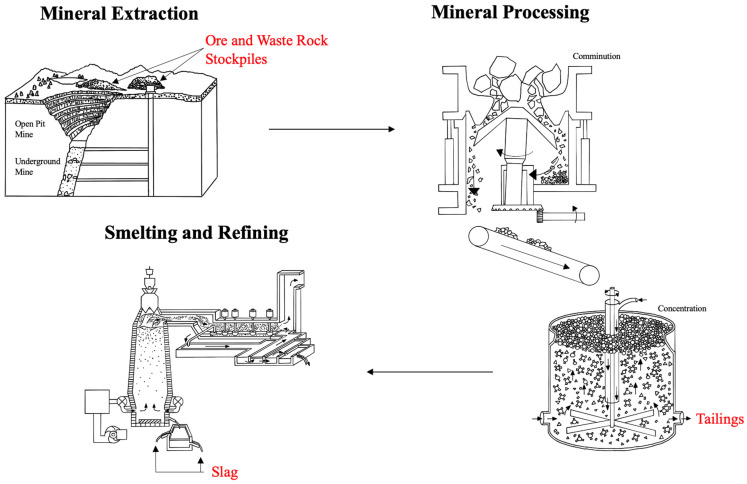
Solid mining waste from mining industry operations, including ore and waste rock stockpiles after mineral extraction, tailings after mineral processing, and slag after smelting. Each of these solid wastes can leach contaminants into the soil and groundwater (adapted from [1,2,3,4]).

Environmental engineering is a broad discipline encompassing a large spectrum of prevention, reduction, recovery, and treatment efforts. While treatment measures are at the bottom of the environmental management hierarchy, they are essential to the longevity and prosperity of the geo-environment. Remediation refers to a reversal of or reduction in environmental damage. This includes waste management from anthropogenic activities, including mining operations. Mining waste from both dated (orphaned and abandoned mines) and developing projects is a significant issue worldwide. Waste is generated from the extraction process (surface and underground mining), mineral processing (comminution, classification, and concentration), and smelting and refining (Figure 1). Waste from each of these operations gets piled in the nearby environment, where it is subject to oxidation and can leach into the surrounding soil and groundwater. As a result, it can cause metal(loid) contamination that is highly acidic or highly alkaline, causing risk to the nearby environment and ecosystems.

Mining is an essential part of society necessary to maintain the quality of life for a vastly growing population and to ensure development and progress. While mining has progressed over the years to consider and incorporate environmental approaches, the severe contamination produced by current and historical mine activities has created considerable ecological damage. Mine-related contamination is often derived from the poor management of stockpiled ore and waste rock, tailings, and slag dumps. These waste piles are impacted by changing environmental conditions. Redox changes, for example, can increase the toxicity and/or mobility of contamination, causing leachate to migrate to soil, groundwater, and surface water sources. Since there is a continued demand for minerals and over-extraction ensures more complex mining operations, it is likely that the volume of waste will increase with decreased ore cut-off grades [5]. This will increase the likelihood of leachate contamination. Remediation is therefore essential to the mitigation of point source contamination from mining activities. It is important that these contaminated sites be monitored and managed to reduce the fate and transport of metal(loid)s, which impact the environment and can deteriorate human and animal health.

Remediation, specifically bioremediation, is crucial for the green and sustainable treatment of mining waste. Many environmental engineering technologies have high costs, long response times, and/or non-compliant leachate rates or metal concentrations post reclamation. Biological remediation techniques have the potential to mitigate the environmental impact experienced during cleanup and pose an advantageous solution for the field of remediation. MICP is a green and sustainable geotechnical engineering technique. The method facilitates chemical precipitation with various microorganism species to precipitate solid CaCO_3_ polymorphs, creating a biocement matrix. Other terms used synonymously for MICP include biocementation, bacterial carbonatogenesis, and biocalcification. The aim is to remove, transform, or immobilize metal(loid) contamination. The overall objective of this research is to demonstrate MICP capability and the efficacy of the bioremediation technique, specifically with respect to metal(loid) immobilization and leachate reduction. This review evaluates MICP as a method for solid waste remediation derived from mining operations.

## 2. Biochemical Processes

### 2.1. MICP

MICP is a biological enhancement to chemical precipitation processes. The reactions are biochemical, utilizing microorganisms as a catalyst to facilitate precipitation. As with chemical precipitation, the reactions are thermodynamically and kinetically driven. Precipitation is governed by a thermodynamic state of instability, whereby the solute concentration exceeds the liquid–solid equilibrium (supersaturation state) causing precipitates to form [6,7]. Kinetically, these precipitates develop through nucleation, growth, and agglomeration, referring to the birth and enlargement of particles [7].

Biologically, this process can occur naturally using indigenous microorganisms (biostimulation), or with the addition of exogenous microorganisms (bioaugmentation). Indigenous microorganisms can adapt to their environment and develop a resistance to the toxicity of the contamination, specifically creating a tolerance to high metal(loid) concentrations [8,9]. However, if microorganisms are not naturally present or a specific strain is not available, exogenous microorganisms can be added to facilitate the process. In both scenarios, the microorganisms require nutrients and energy to stimulate growth. The application of a nutrient broth (NB) is required to enhance microbial survival, especially within nutrient-deficient mining waste. The microorganisms precipitate solids through biologically mediated mechanisms (passive precipitation caused by organic matter from microbial activity), biologically controlled mechanisms (direct precipitation from cellular activity), or biologically induced mechanisms (direct precipitation resulting from environmental changes caused by biological activity) [10,11].

MICP is a biologically induced mechanism. It occurs from direct biological activity that alters the extracellular environment. These microbial pathways include enzyme activity, oxidation–reduction reactions, or photosynthesis processes. Microorganisms can release organic acids, electron donors, and enzymes into the extracellular environment through passive diffusion, secretion, and active pumping [11,12,13,14]. Again, this impacts the extracellular environment, which can induce precipitation.

Table 1 outlines the different types of MICP and the various subcategories of microbial pathways. The microbial enzymes urease and carbonic anhydrase (CA) facilitate CaCO_3_ precipitation from urea hydrolysis and carbon dioxide (CO_2_) hydration, respectively. These enzymes can work together to precipitate CaCO_3_, since urease converts urea ((NH_2_)_2_CO) into ammonia (NH_3_), then ammonium (NH_4_^+^), and CA converts carbonic acid (H_2_CO_3_) to bicarbonate (HCO_3_^−^), then carbonate (CO_3_^2−^) [11]. However, CA can also precipitate CaCO_3_ independently. It can act as a catalyst for the transformation of atmospheric CO_2_ into MCO_3_ compounds, which can enhance precipitation [15]. The redox-driven forms of MICP (denitrification, sulfate reduction, iron reduction, methane oxidation, and ammonification [16,17]) utilize oxidative–reductive reactions to enable precipitation. The redox transformations alter the solubility [18,19], which alters the saturation and supersaturation states, leading to precipitation [20]. Finally, photosynthetic bacteria (cyanobacteria and microalgae [11]) facilitate precipitation via the synthesis of atmospheric CO_2_ into organic matter and simultaneous HCO_3_^−^/OH^−^ exchange across the cell membrane, followed by CaCO_3_ precipitation within the cell (excess Ca^2+^ is stored in the cell membrane), or extracellularly via an antiporter [17]. In all situations, precipitation favors high pH conditions.

### 2.2. Mining Waste Characterization and Treatment

The type of MICP mechanism utilized will be dependent on the indigenous microorganisms at the site (i.e., biostimulation) and the type of waste. With respect to pH of the leachate generated, mining waste can be categorized into three groups: acidic, neutral, and alkaline waste. During mineral extraction and processing, overburden material is stockpiled, and tailings are discharged into tailing storage facilities (TSFs). Tailings typically consist of ore residues which can leach into the soil and groundwater, causing contamination. The leachate behavior is based on the chemistry of the infiltrating water and receiving water, the composition and age of the materials including co-deposited wastes, and the geophysical site (topography, soil porosity and permeability, flow rates, redox potential, etc.) [25]. This water balance will influence the fate and transport of soluble contaminants, such as metal(loid)s, in mining waste. In this study, only acid and alkaline mine-generated waste will be discussed.

(1) $$4{FeS}_{2}+15O_{2}+14H_{2}O\to 4{Fe(OH)}_{3}+8{SO}_{4}^{2-}+16H^{+}$$

Acid rock drainage (ARD), or acid mine drainage (AMD), refers to contaminated, acidic drainage water, typically originating from mines or mining activities. The generation process of AMD involves the oxidation of sulfur or sulfide (S^2−^) minerals, whereby Equation (1) shows the oxidation of pyrite (FeS_2_) into ferric hydroxide (Fe(OH)_3_) and sulfuric acid (SO_4_^2−^ and H^+^) [26]. AMD is characterized by low pH and high concentrations of sulfates and metal(loid)s [27]. The rate of AMD generation is dependent on sulfide morphology, oxygen content, wetting/drying cycles, microbial activity, and geologic history [28]. Sulfate reduction via sulfate-reducing bacteria (SRB) is a prevalent method to treat AMD using MICP. As shown in the equation in Table 1, a carbon source (electron donor) facilitates the dissimilatory sulfate reduction from SO_4_^2−^ to H_2_S [24]. The reaction releases CO_2_ and hydroxide ions (OH^−^) which are utilized in the formation of solid CaCO_3_.

(2) $$CaO+H_{2}O↔{Ca(OH)}_{2}↔{Ca}^{2+}+2{OH}^{-}$$

Alkaline waste is derived from the hydration of alkaline earth oxides. With mine operations, it is often associated with nickel, chrysotile, kimberlite, and red mud mining [29]. With mineral processing operations, it is often associated with gold, alumina, chromite, and uranium [5]. Equation (2) demonstrates a generic chemical reaction utilizing calcium oxide (CaO), although magnesium oxide (MgO), sodium oxide (Na_2_O), and ferrous oxide (FeO) are also common [25,29,30]. Alkaline waste is characterized by a high content of alkaline earth metals [29], high pH, high salinity, high sodicity, and fine particle size [5]. Alkaline waste from mining leachate can cause high pH, high chemical oxygen demand, oxygen depletion, high sulfate loadings, salinity, and high concentrations of metal precipitates [25].

(3) $$H_{2}{CO}_{3}+{Ca(OH)}_{2}↔{CaCO}_{3}+2H_{2}O$$

(4) $$CaSiO_{3}+{CO}_{2}\to {CaCO}_{3}+{SiO}_{2}$$

Alkaline waste can be treated using MICP via microbial activity from the CA enzyme, which transforms CO_2_ to H_2_CO_3_. The H_2_CO_3_ will react with the calcium hydroxide (Ca(OH)_2_) from Equation (2) to produce CaCO_3_, as shown in Equation (3) [30]. In addition, carbonate precipitation can mimic the natural weathering process of silicate minerals [25,29]. This is shown in Equation (4), whereby the dissolution of alkaline earth metals from the silicate matrix is required for carbonate precipitation. This can occur as a direct process (occurs in one step: dissolution and precipitation) or an indirect process (occurs as two steps: dissolution via lixiviate, then precipitation) [25,31]. The carbonate precipitation of alkaline waste is impacted by the solid-to-liquid ratio, particle size, and temperature [25].

The type and characteristics of mining waste will influence the MICP design. MICP can be utilized as a remediation strategy to treat metal(loid) contamination that is highly acidic or alkaline. The geophysical mechanisms involved in the remediation of solid mining waste (waste stockpiles, tailings, and slag dumps) are detailed as follows.

## 3. Bioremediation Processes—Geophysical and Biochemical Interactions

### 3.1. Bioremediation

**Figure 2:**
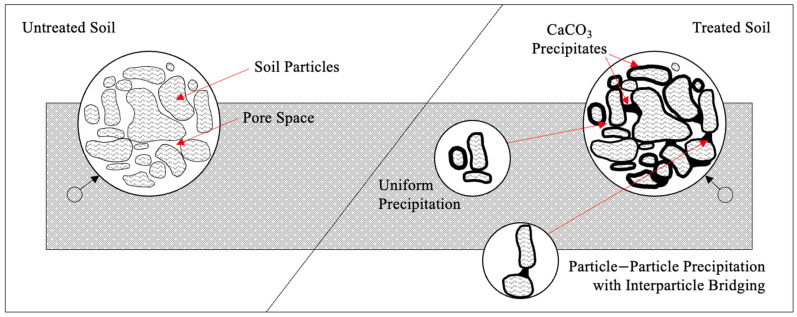
Untreated and MICP-treated soil demonstrating uniform and particle–particle precipitation creating a biocement matrix (adapted from [24,32]).

Precipitation (and/or co-precipitation (CaCO_3_/MCO_3_)) creates a biocement matrix formed by the interconnection of CaCO_3_ precipitates. At the microscale (via microbial activity), precipitates form uniformly around soil particles or between particle–particle contacts (Figure 2). Effective bridges are formed at the pore throats due to capillary force. In both scenarios, the precipitates decrease pore space and reduce hydraulic conductivity [32]. They also reduce soil void volume and increase soil cohesion, which decreases permeability and causes a plugging effect [33]. Further, particle–particle precipitation and the creation of effective bridges is speculated to improve soil, specifically shear strength [32].

MICP remediates metal(loid) contamination through removal, immobilization, impermeable barriers, and liquefaction reduction. The removal of metal(loid) contamination is due to the direct precipitation of CaCO_3_ and MCO_3_ minerals, while immobilization is attributed to the decrease in leachate caused by MICP [33]. The reduction in leachate is often assessed through the decrease in the soluble–exchangeable fraction, which indicates contaminant bioavailability and mobility [18,22,34,35,36]. Leachate is also reduced by the surface deposition and clogging of pore spaces via CaCO_3_ crystals [21,37]. The development of the biocement matrix will create an impermeable barrier by establishing a plugging effect [38,39]. For specificity, remediation via MICP can be direct (fixed in CaCO_3_ or MCO_3_ precipitates [35,39,40,41]) or indirect (via metal(loid)-CaCO_3_ complexes [21,22,42], inclusion in the crystal structure [35,40,42], and/or sorption [35,39]). The inclusion of metal(loid)s into the crystal structure occurs from divalent cations similar to Ca^2+^ ions (i.e., ion radius and ion charge), which are integrated into the crystal matrix by substitution/ion exchange, or are integrated via fissures and/or interstices [42]. Divalent cations that are easily substituted through ion exchange are shown in Table 2. Further, sorption can refer to the adsorptive properties of CaCO_3_, which has been used as an adsorbent for metal(loid) removal [43,44].

Again, the surface deposition of CaCO_3_ and precipitation within the pore spaces will create a plugging effect or impermeable barrier. The precipitation of CaCO_3_ crystals at the surface of waste piles can negate physical degradation (via wetting and drying (W/D), freezing and thawing (F/T), hot and cold (H/C), wind, percolating fluids, erosion, physical loading stresses, etc.) [22,47,48,49]. This crust can decrease water absorption and permeability through the specimen [48,50], reducing metal(loid)-contaminated leachate [21,37]. The precipitation of CaCO_3_ crystals within the pore spaces will also decrease permeability, while simultaneously increasing strength [22,50]. Comprehensive strength is increased through the consolidation of biologically induced CaCO_3_ precipitates [50], whereby precipitates form in the pore spaces within sample fractures and fissures. This can reduce liquefaction, specifically tailing liquefaction, since the biocement matrix (i.e., particle–particle precipitates) reduces pore water pressure [33].

Microorganisms, once introduced to the system, will transport and adsorb to solid particles [51], acting as a location for nucleation and growth [14]. The bacterial cell wall has a negative surface charge due to carboxyl, phosphoryl, amino, and sulfo groups [13,52]. This can attract heavy metals and metalloids, causing adsorption, redox changes, and precipitation [53]. In addition, extracellular polymeric substances (EPSs) and biofilm formation can reduce pore size, hydraulic conductivity, and permeability [54], again decreasing fluid movement through the contaminated area. Precipitation at the biofilm boundary can coat pore space and individual grains [21]. The EPSs from microorganisms have strong metal(loid)-binding capacity [46], and metal(loid) ions can adsorb on functional groups of EPSs [20]. This means metal(loid) contaminants in soil and groundwater may be immobilized via microbial EPS secretion. While this is not the direct effect of MICP (a biologically induced mechanism), it is considered a biologically mediated mechanism [10,11]. Passive CaCO_3_ precipitation occurs when a pH increase causes functional groups to deprotonate, creating a negative charge of EPS, and leading Ca^2+^ ions to bind and precipitate [11].

**Figure 3:**
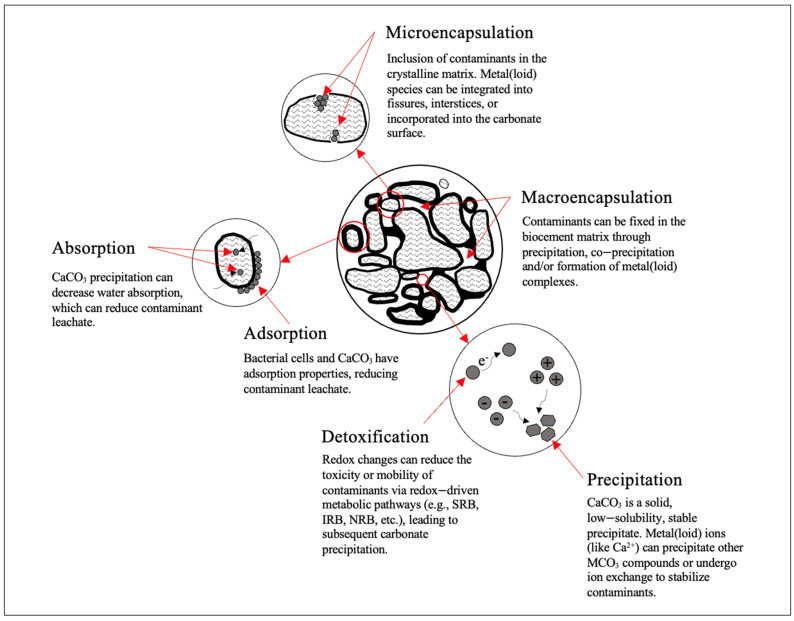
Mechanisms of bioremediation via MICP and details of the immobilization of fixation of metal(loid) contaminant species.

As a biological remediation technique, MICP is bio-physiochemical process whereby metal(loid) species are immobilized or fixated at the site (in situ). The mechanisms attributed to its efficacy as a remediation strategy are detailed in Figure 3. These mechanisms (macroencapsulation, microencapsulation, absorption, adsorption, precipitation, and detoxification) are inspired by the well-established solidification/stabilization technique [47], whereby MICP broadly mimics the immobilization mechanisms [55]. However, this process is not without disadvantages. Metal(loid)s can be released or redissolved with physical degradation, physiochemical stress, or changes to their environmental conditions (i.e., pH, redox conditions).

### 3.2. MICP Indicators

Prior to MICP application, a thorough sample analysis is required to characterize the tailings or metallurgical waste. This will include physical (particle size distribution, porosity, permeability, density, moisture content, etc.), chemical (pH, organic content, metal(loid) content, cation exchange capacity (CEC), salinity, electrical conductivity, etc.), biological (microorganism extraction, enzyme activity, etc.), and mineralogical examination.

The application of MICP can vary based on the site characteristics. These applications can be in situ or ex situ operations, dependent on the project specifications. Prominent in situ operations include injection or surface percolation. However, both have drawbacks including clogging and uneven distribution, leading to inhomogeneous CaCO_3_ precipitation. As an ex situ treatment, pre-mixing solves these issues. Although, disturbance of the solids can create pseudo stress that complicates precipitation [32].

The main indicators to assess the efficacy of MICP as a bioremediation technique for mining waste are strength (i.e., mechanical strength, slaking behavior, and water absorption) and leach resistance (i.e., pH, mineralogy, and hydraulic conductivity) [37,55]. Table 3 defines indicators and tests that can be used to assess CaCO_3_ precipitation and its applicability as a remediation technique.

(5) $$\frac{Q}{A}=k\sqrt{t}$$

As mentioned, the surface deposition of biologically precipitated CaCO_3_ and precipitation within the pore spaces will decrease water absorption [22,50,57], leading to decreased permeability [58]. As CaCO_3_ precipitates on the sample surfaces and in between pore spaces, the material should resist water penetration, since fluid passage ingress is reduced. This will reduce contaminated leachate in soil and groundwater sources [22]. It will also diminish the degradation processes of MICP-treated samples [22,50]. The sorptivity test, specifically, measures the tendency of a material (e.g., tailings) to absorb water via capillarity [15]. The sorptivity is calculated using Equation (5), where Q/A is plotted against √t and k is determined by the slope of the linear relationship [15,22,50]. Q is the water absorbed in cm^3^, A is the cross-sectional area in cm^2^, k is the sorptivity in cm/s^1/2^, and t is the time in s [50].

(6) $$K=\rho \frac{H}{P}\frac{Q}{A}$$

For application, the reactive medium (nutrients, microorganisms, CO_2_, etc.) need to penetrate the entirety of the sample to establish uniform and homogenous MICP. For optimal application, the surface percolation and/or injection rates should be analyzed. However, after MICP, both the permeability and hydraulic conductivity should decrease. Water permeability relates to the permeability coefficient [56]. Therefore, the permeability coefficient and the water impermeability are inversely proportionate. MICP prefers a strong impermeability and low permeability coefficient value. Impermeability relates to porosity and pore spaces. As CaCO_3_ precipitates, the pore spaces will decrease, leading to a decrease in water ingress and an increase in impermeability. With a slower water flow rate, soil and groundwater leachate will likely minimize. The permeability coefficient (K) can be calculated using Equation (6), where K is the permeability coefficient in cm/s, ρ is the density of water in kg/cm^3^, H is the length of sample in cm, P is the water pressure in kg/cm^2^, Q is the net rate of inflow in cm^3^/s, and A is the cross-sectional area in cm^2^ [59].

To further assess the plugging effect of MICP, slaking behavior using the slake test can be used. This identifies the long-term implication of weather and erosion on CaCO_3_ precipitates and the biocement matrix. There is often a tendency for large materials to break down or disintegrate when subjected to W/D. If materials contain clays, there may be a swelling effect when wetting is followed by contraction when drying, creating cracks and other defects.

A thorough approach to analyze the bioremediation of MICP-treated mining waste has been completed [37]. The experiment tested unconfined compressive strength (UCS), water absorption, slaking behavior, hydraulic conductivity, and leachability. The study analyzed two scenarios whereby NB and urea were applied to replicate a saturated condition (e.g., immersed) and a drained condition (e.g., flow through). The UCS of the immersed condition had high UCS at the top of the sample which decreased significantly toward the middle and bottom of the samples, likely due to accumulation of the cementation solution at the injection point causing a subsequent clogging effect. This is in opposition with the flow-through condition, which had a lower UCS at the top of the sample but higher UCS throughout, likely due to the varied saturation states and CaCO_3_ precipitation throughout the sample. The water absorption and hydraulic conductivity of treated MICP samples decreased from the controls, indicating a reduction in water infiltration and reduction in leaching rates. The slaking behavior demonstrated a resistance to slaking in MICP-treated samples where controls showed none, signifying a resistance to weather and erosion impacts. Further, the leachability showed a significant decrease in Pb concentration after MICP treatment, revealing a decrease in Pb migration out of the solid phase, essentially preventing toxic, water-soluble Pb leachate. The XRD, SEM, and EDS analyses all showed an increase in calcite in MICP-treated samples where CaCO_3_ precipitates bound the grains, immobilizing Pb within the biocement structure and reducing solubility and toxicity [37].

With respect to leachability, the five-stage Tessier sequential extraction method can be used to assess fractionation (exchangeable, carbonate-bound, iron and manganese oxide-bound, organic-bound, and residual fractions) of MICP-treated samples [35,60]. The results can be used as an indicator for biologically influenced precipitation. If the exchangeable fraction in control specimens far exceeds the MICP-treated samples for the desired metal(loid), the biological remediation of mine waste is considered feasible. In addition, the increase in carbonate-bound fraction between the control and MICP-treated samples indicates CaCO_3_ precipitation is attributed to biological measures. This experiment can be used as evidence of MICP presence and potential remediation capability. However, the experiment gives variable, inconsistent results, and should only be used to demonstrate MICP feasibility.

Biostimulated microorganisms from Sr-contaminated mine tailings were applied to an aquifer quartz sand to test leachability [40]. The soluble–exchangeable fraction and the carbonate-bound fraction of the five-stage Tessier sequential extraction method were analyzed against a control. While the soluble–exchangeable fraction decreased, the carbonate-bound fraction increased, indicating bacterial remediation. XRD analysis found inorganic CaCO_3_ precipitation (calcite) in the control specimens; however, there were significantly more CaCO_3_ precipitates (calcite, aragonite, and vaterite) in the bioremediated samples. Furthermore, the SEM and XRD analyses established the co-precipitation of calcite–strontianite (SrCO_3_) solids [40].

Other leaching tests include column leaching and humidity cell tests. The column leaching test is a static leaching method that can be used to analyze the mobility of contaminants from MICP-treated samples [22]. Column leaching will provide insight into the leachability of the prepared samples, and the leachate solution can also be analyzed to assess metal(loid) concentration (e.g., via inductively coupled plasma mass spectrometry (ICP-MS) and atomic absorption spectrometry (AAS)). The humidity cell test is a kinetic leaching test common to the mining industry known as a geochemically reliable method [61]. It is a long-term test (>20 weeks) that floods and aerates the sample to mimic weathering and provides insight into the generation of acidic, alkaline, or neutral effluent, and inorganic constituents released as leachate [62]. In addition to these tests, the pH value of leachate solutions can be used as an indicator for MICP.

Mining waste leachability prior to and post MICP treatment to determine feasibility as a remediation strategy has been examined [21]. The results were variable, suggesting further experimentation and MICP optimization. Both the quantity and quality of leachate were assessed. High quantities of leachate were experienced post MICP in course-grained samples, indicating an inability to clog pore spaces. A thicker, homogenous layer of CaCO_3_ precipitation is required to reduce permeability and therefore leachate. It is likely that MICP would be suited to a fine-grained media (i.e., mine tailings or metalliferous waste). The quality of leachate was also thoroughly investigated. Metal(loid) concentrations (i.e., Cd, Pd, Zn) post MICP treatment were reduced, signifying immobilization. However, in most samples As concentrations increased, likely due to the solubility increase in oxy-anions (i.e., arsenic oxyanions) with pH increases. The concentration of Cu in leachates was also varied. Some samples experienced an increase in Cu concentration likely due to the formation of CO_3_^2−^complexes (specifically Cu–ammonia complexes (i.e., Cu(NH_3_)_4_^2+^ and Cu(NH_3_)_3_^2+^), which have high mobility). The presence of high mobility (with high pH) oxy-anions should be evaluated prior to MICP treatment. Further, the reduction in NH_3_ by-product from urease MICP may reduce mobile ammonia complexes formed during treatment [21].

Other MICP evaluation techniques include the utilization of analysis tools [29]. The most common are the following:

- Scanning electron microscopy (SEM): This is used to create clear images of MICP-treated samples. It shows precipitates and bacteria species (including shapes), which will better illustrate CaCO_3_ precipitated by bacterial influence.
- Energy-dispersive X-ray spectroscopy (EDS): This method establishes the proportion of elements that make up a sample. It will provide a comparison between control and MICP-treated samples, which will illustrate the precipitates formed (i.e., CaCO_3_) and the co-precipitation of different metal(loid)s.
- X-ray diffraction (XRD): This is used to verify precipitates formed during MICP. It confirms CaCO_3_ (noting specific polymorphs present) and the co-precipitation of metal(loid)s, which will create a better understanding of the mechanisms involved in the bioremediation of mining waste.

### 3.3. Case Studies

MICP is typically studied in terms of geotechnical engineering. Its application is diverse, and includes the restoration of calcareous stones and construction materials [17,57,63,64], concrete strengthening [57,63,64,65], soil strengthening [51,66,67], selective plugging for oil recovery [17,58,63,68], bio-clogging [51,69,70], the enhancement of soil thermal conductivity [51,71,72], dust suppression [33,51,73,74], erosion control [49,51,54,75,76,77], liquefaction mitigation [33,51,54,75,78,79], wastewater treatment [63,80], CO_2_ sequestration [17,54,81,82], and bioremediation [34,36,83,84,85,86]. While applications for soil and cement improvement typically utilize bioaugmentation, bioremediation often utilizes biostimulation [51]. Biostimulation is used due to the toxicity of waste since indigenous microorganisms can adapt and develop a resistance to high concentrations of essential and nonessential metal(loid)s [8].

The most common metabolic pathway for MICP bioremediation is ureolysis [17]. Urea hydrolysis using ureolytic bacteria is considered optimal since the microorganisms exist in a wide range of environments [16], there is a high conversion to CaCO_3_ precipitation [24,87], the process is easily controlled [24,87,88], the timeframe is short [87,88,89], the cost is low [16], and it is not sensitive to redox changes [42,46]. Popular ureolytic microorganisms in the literature include *Sporosarcina pasteurii* (previously known as *Bacillus pasteurii*) and species from the genus *Bacillus* (e.g., *Bacillus sphaericus*, *Bacillus megaterium*, *Bacillus subtilis*, *Bacillus mucilaginous*, *Bacillus lentus*, etc.) [17,89]. Table 4 demonstrates several studies looking specifically at metal(loid) bioremediation using urease-driven MICP. These studies illustrate the promising potential of MICP as it relates to mine waste remediation.

Evident from Table 4 is the increased use of biostimulation compared to bioaugmentation. Microbial growth and enumeration are necessary to achieve adequate MICP efficacy within toxic conditions. Typically, this is facilitated with an NB (mixture of nitrogen, carbon, phosphorus, etc.) and a cementation solution (urea and CaCl_2_). As marked in Table 4, these nutrient mediums are case-specific and should be optimized accordingly. However, it should be noted that secondary contamination from nitrogen, phosphorus, etc., may occur depending on the quantity of NB and microbial utilization.

The experiments in Table 4 illustrate the capacity with which MICP can act as a bioremediation method for metal(loid) contamination for altering the biochemical and geophysical aspects of mine tailings or metalliferous waste. The mechanisms of immobilization include the direct precipitation of CaCO_3_, the co-precipitation of MCO_3_, metal(loid) inclusion into CaCO_3_ crystals, adsorption, metal complexation, and precipitation with functional groups causing reduced permeability, water absorption, hydraulic conductivity, slaking, and increased strength. While some studies focused more on the biochemical components illustrating MICP feasibility within mining waste [35,40,41,42], others focused more on the geophysical component, addressing MICP practicality as a bioremediation strategy [21,37,38,39]. The variety of immobilization mechanisms interpreted from these studies demonstrates MICP’s versatility in the field of bioremediation. Current research investigates MICP application to tailings [90,91,92,93], deep-sea mining [94], and contaminated soil nearby mining and smelting operations [95,96].

## 4. Recommendations

There remains significant opportunity for research with regards to MICP as a bioremediation method, specifically as it relates to mining waste. Additional experimentation at the laboratory scale, pilot scale, and field scale are required to demonstrate its practicality and feasibility as an environmental remediation technique. The following gaps in the literature require further investigation:

- Long-term feasibility: additional research is required to evaluate MICP over long timeframes in practical field scenarios to establish its long-term feasibility. Some considerations impacting long-term feasibility include:
  - Annual climate changes may impact the long-term feasibility of the CaCO_3_ precipitates and the biocement matrix. Testing climate change (e.g., temperature and moisture content changes) will better indicate year-round feasibility as a remediation strategy. This can be expanded to an assessment of F/T cycles and W/D cycles on MICP formation and overall efficacy. Further, CaCO_3_ solubility is impacted by temperature and carbon dioxide (CO_2_) concentration [97]. As temperature increases, solubility decreases [97,98], while solubility increases with CO_2_ increases [97]. These factors may impact the saturation state and therefore MICP-driven precipitation.
  - The dissolution of immobilized contaminants over time:
    - pH changes: MICP forms geochemically stable CaCO_3_ precipitates [46]. However, little long-term research has been conducted on the process. A fundamental factor governing metal precipitation is pH. Alkaline solutions are more likely to cause precipitation, while acidic solutions can cause the dissolution of metal precipitates [99]. Therefore, changes in pH could potentially redissolve metal precipitates.
    - Redox potential changes: The speciation state can influence oxidation, reduction, mineralization, and immobilization [100]. Certain metal(loid)s are more stable under reducing conditions or oxidizing conditions. Therefore, redox changes can cause the dissolution of precipitates over time. While urease-driven MICP is not readily influenced by redox potential [42], sulfides and iron oxides are easily reactive with redox potential changes [46]. It is possible that immobilized metal(loid)s can be redissolved [46]. The impact of chemical speciation and redox changes should be studied with respect to time to assess their long-term impact.
    - Biocement defects: Metal(loid)s immobilized by MICP can release and leach into the soil and/or groundwater via cracks, fissures, or interstices developed in the biocemented matrix. Over time, physical degradation from weather may cause defects causing immobilized contaminants to leach back into the environment. Again, a long-term assessment of MICP is required to establish its practicality as a bioremediation technique.
- Secondary contamination and by-products: A better understanding of secondary contamination and potential by-products is required for a practical field application of MICP. In addition to identifying the presence of these contaminants, mitigative strategies require exploration to minimize their effect.

## 5. Conclusions

MICP is a promising engineering technique. As a geotechnical engineering strategy, MICP has achieved documented success and demonstrated its feasibility as a promising method at the laboratory scale. Current research is examining MICP as a bioremediation strategy for metal(loid) contamination with potential interest for the management of waste in the mining industry. The method is applicable to many scenarios utilizing various microorganisms and microbial pathways. As a bioremediation strategy, MICP can immobilize contaminants via direct and indirect processes. Although there are currently gaps in the literature, MICP offers an innovative solution for remediation that is both positive in a socio-economic sense and eco-friendly. Additional research is required prior to long-term field application to reduce the reactivation of immobilized metal(loid)s and minimize/mitigate secondary contaminants and by-products.

## Figures and Tables

**Table 1:** Type of MICP-driven reactions [16,17].

Energy Pathway	Type	Microbial Pathway	Microbial Group	Reactions *	References
Heterotrophic	Enzyme-driven	Urea hydrolysis (urease enzyme)	Ureolytic bacteria	${\left({NH}_{2}\right)}_{2}CO+2H_{2}O\to 2NH_{3}+H_{2}{CO}_{3}$ $2NH_{3}+2H_{2}O↔2NH_{4}^{+}+2{OH}^{-}$ $H_{2}{CO}_{3}+2{OH}^{-}↔CO_{3}^{2-}+2H_{2}O$ $Bacteria+{Ca}^{2+}\to Bacteria-{Ca}^{2+}$ $Bacteria-{Ca}^{2+}+CO_{3}^{2-}\to Bacteria-{CaCO_{3}}_{\left(s\right)}↓$	[21,22]
		CO_2_ hydration (CA enzyme)	Genetic families (α-, β-, and γ-classes)	${CO}_{2}+H_{2}O↔H_{2}{CO}_{3}↔{HCO}_{3}^{-}+H^{+}$ ${Ca}^{2+}+2{HCO}_{3}^{-}\to {CaCO_{3}}_{\left(s\right)}↓+{HCO}_{3}^{-}+H^{+}\to {CaCO_{3}}_{\left(s\right)}↓+{CO}_{2}+H_{2}O$	[15,23]
	Redox-driven	Denitrification	NRB	${NO}_{3}^{-}+\frac{5}{4}{CH}_{2}O\to \frac{1}{2}N_{2}+\frac{5}{4}{CO}_{2}+\frac{3}{4}H_{2}O+{OH}^{-}$ ${Ca}^{2+}+{CO}_{2}+2{OH}^{-}\to {CaCO_{3}}_{\left(s\right)}↓+H_{2}O$	[24]
		Sulfate reduction	SRB	${{SO}_{4}^{2-}+2CH}_{2}O\to H_{2}S+2{CO}_{2}+2{OH}^{-}$ ${Ca}^{2+}+{CO}_{2}+2{OH}^{-}\to {CaCO_{3}}_{\left(s\right)}↓+H_{2}O$	[24]
		Iron reduction	IRB	$4{Fe}^{3+}+{CH}_{2}O+H_{2}O\to 4{Fe}^{2+}+{CO}_{2}+4H^{+}+{Ca}^{2+}+{HCO}_{3}^{-}+{OH}^{-}\to {CaCO_{3}}_{\left(s\right)}↓+H_{2}O$	[16]
		Ammonification	Myxobacteria	$Amino\;Acid+O_{2}\to NH_{3}+{CO}_{2}+H_{2}O$ $NH_{3}+H_{2}O\to NH_{4}^{+}+{OH}^{-}$ ${CO}_{2}+{OH}^{-}\to {HCO}_{3}^{-}$ ${Ca}^{2+}+{HCO}_{3}^{-}\to {CaCO_{3}}_{\left(s\right)}↓+H^{+}$	[17]
Heterotrophic or autotrophic		Methane oxidation	Methanogens	${CH}_{4}+{SO}_{4}^{2-}\to {HS}^{-}+{HCO}_{3}^{-}+H_{2}O$ ${Ca}^{2+}+{HCO}_{3}^{-}\to {CaCO_{3}}_{\left(s\right)}↓+H^{+}$ $H^{+}+{HS}^{-}\to H_{2}S$	[17]
Autotrophic	Photosynthesis-driven	Photosynthesis	Cyanobacteria algae	${CO}_{2}+H_{2}O\to {CH}_{2}O+O_{2}$ ${HCO}_{3}^{-}\to {CO}_{2}+{OH}^{-}$ ${Ca}^{2+}+2{HCO}_{3}^{-}\to {CaCO_{3}}_{\left(s\right)}↓+{CH}_{2}O+O_{2}$	[17]

* By-products noted in red.

**Table 2:** Divalent cations for MICP ion exchange [42,45,46].

Ion	Abbreviation	Charge	Calculated Radius (pm)	Series
Calcium	Ca	+2	194	Alkaline Earth Metals
Strontium	Sr	+2	219	Alkaline Earth Metals
Lead	Pb	+2	154	Post-transition Metal
Cadmium	Cd	+2	161	Transition Metal
Copper	Cu	+2	145	Transition Metal

**Table 3:** MICP indicators and tests.

Indicator	Definition	Test
Permeability and hydraulic conductivity	Provides information on flow rate through the materials (e.g., tailings), whereby a larger permeability coefficient means fluids are flowing rapidly through the tailings [56].	Water permeability
Comprehensive strength	This is the capacity by which a material (e.g., tailings) can withstand a load.	The oedometer test
		The direct shear box test
		The triaxial test
		The pocket cone penetrometer test
		The needle penetration test
Water absorption	Testing is used to examine the resistance toward water penetration (e.g., rainfall, capillary rise in groundwater, and slope runoff [37]).	The sorptivity test
Leaching tests	These tests are required to demonstrate a reduction in leachate quantity and a reduction in contaminant concentration.	The five-stage Tessier sequential extraction method
		Column leaching
		Humidity cell tests
Slaking behavior	This is a physiochemical property establishing material impact to W/D cycles common to external environments. It can also be used to indicate resistance to erosion.	The slake test

**Table 4:** MICP as a bioremediation technique for metal(loid) immobilization utilizing urease-producing microorganisms.

Reference	Sample		Microorganism	Nutrient Medium	Speculated Mechanism of Immobilization
	Sample Type	Target Metal(loid)			
[42]	Mining area near Urumqi, China	Cu	*Kocuria flava* ^1^	NB ^3^, cementation solution [urea (2%), CaCl_2_ (25 mM)]	-Involvement of functional groups. -Metal complexation. -Incorporation into CaCO_3_ crystal.
[41]	Mining area near Urumqi, China	Pb	*Kocuria flava* ^1^	NB ^3^, cementation solution [urea (2%), CaCl_2_ (25 mM)]	-PbCO_3_, PbO, PbO_2_, Pb_3_(CO_3_)_2_(OH)_2_ precipitates. -Transformation of Pb into geochemically stable calcite.
[40]	Mine tailings from Xinjiang Uyghur Autonomous Region, China	Sr	*Halomonas* sp. ^1^	NB [Peptone (10 g/L), beef extract (1.5 g/L), yeast extract (1.5 g/L), NaCl (5 g/L)], cementation solution [urea (2%), CaCl_2_ (25 mM)]	-Sr co-precipitation with CaCO_3_ (SrCO_3_) via substitution or inclusion. -Calcite–strontianite solid precipitates.
[35]	Mine tailings from Jeongeup, Jeollabuk-do, Korea	Pb	*Bacillus* sp. ^1^	LB agar plates	- Pb(NO_3_)_2_ conversion into PbS and PbSiO_3_. -CaCO_3_ precipitation. -Absorption of Pb onto CaCO_3_ precipitates. -Ca^2+^ substitution with Pb^2+^ in CaCO_3_ lattice. -Pb inclusion through interstices and defects in CaCO_3_ precipitates.
[21]	Carpenter Snow Creek Mining District in the Little Belt Mountains near Neihart, Montana	As Cd Pb Cu Zn	95.15% *Sporosarcona* ^1^ *&* 2.75% *Acidovorax* ^1^	NB ^3^ (10 mL/L), yeast extract (0.5 g/L), urea (10 g/L), cementation solution [yeast extract (0.5 g/L), urea (20 g/L), CaCl_2_*2H_2_O (49 g/L)]	-CaCO_3_ precipitation. -CO_3_^2−^ complexes (i.e., Cu–carbonate complexes which decrease Cu^2+^ toxicity). -Co-precipitation of metals.
[39]	Mining area in Gangwondo, Korea	Cd	*Lysinibacillus sphaericus* ^1^	NB [Beef extract (3 g/L), peptone (5 g/L), urea (20 g/L), micro agar (10 g/L) containing cycloheximide (100 μg/mL)], cementation solution [CdCl_2_·5H_2_O (50 mM)]	-CaCO_3_ and CdCO_3_ precipitation. -Cd adsorption onto calcite surface. -Ca-Cd solid solution at the surface (i.e., surface crust).
[38]	Abandoned metal mine sites in Gangwondo, Korea	Pb	*Enterobacter cloacae* ^1^	NB [Beef extract (3 g/L), peptone (5 g/L), urea (20 g/L), micro agar (10 g/L) containing cycloheximide (100 μg/mL)], cementation solution [PbCl_2_ (1 M)]	-Reduced permeability caused by plugging effect of CaCO_3_ precipitates. -PbCO_3_ precipitation.
[37]	Abandoned Kabwe Mine of Central Province, Zambia	Pb	*Pararhodobacter* sp. ^2^	NB [Hipolypeptone (5.0 g/L), yeast extract (1.0 g/L), and FePO_4_ (0.1 g/L)], cementation solution [urea (0.5 M), CaCl_2_ (0.5 M), NaHCO_3_ (0.02 M), NH_4_Cl (0.2 M), and nutrient broth (3 g/L)]	-CaCO_3_ precipitation on the surface and in between sand grains. -CaCO_3_ bridging causing particle binding and reducing pore space. -Decreased water absorption, hydraulic conductivity, and slaking. -Increased material strength. -Pb immobilization within treated samples preventing water-soluble Pb leachate.

^1^ Biostimulation using indigenous microorganisms on the contaminated sample; ^2^ bioaugmentation using exogeneous microorganisms external to the contaminated sample; ^3^ not specified within study; nutrient broth (NB); Luria–Bertani (LB) agar.

## Data Availability

Data are contained within the article.
